# Comparative Characterization of Iron and Silver Nanoparticles: Extract-Stabilized and Classical Synthesis Methods

**DOI:** 10.3390/ijms24119274

**Published:** 2023-05-25

**Authors:** Farida Akhatova, Svetlana Konnova, Marina Kryuchkova, Svetlana Batasheva, Kristina Mazurova, Anna Vikulina, Dmitry Volodkin, Elvira Rozhina

**Affiliations:** 1 Bionanotechnology Lab, Institute of Fundamental Medicine and Biology, Kazan Federal University, Kreml uramı 18, 420008 Kazan, Republic of Tatarstan, Russia; svetaka14@gmail.com (S.K.); maricshka80@gmail.com (M.K.); svbatasheva@gmail.com (S.B.); 2 Department of Physical and Colloid Chemistry, Russian State University of Oil and Gas (National Research University), Leninsky Prospect 65, 119991 Moscow, Russia; mazurovachris55@mail.ru; 3 Bavarian Polymer Institute, Friedrich-Alexander-Universität Erlangen-Nürnberg (FAU), Dr.-Mack-Straße 77, 90762 Fürth, Germany; anna.vikulina@fau.de; 4 Department of Chemistry and Forensics, School of Science and Technology, Nottingham Trent University, Clifton Lane, Nottingham NG11 8NS, UK; dmitry.volodkin@ntu.ac.uk; 5 Department of Biological Education, Institute of Fundamental Medicine and Biology, Kazan Federal University, Kreml uramı 18, 420008 Kazan, Republic of Tatarstan, Russia

**Keywords:** *Sphagnum fallax*, iron nanoparticles (FeNPs), silver nanoparticles (AgNP), extract-stabilized nanoparticles

## Abstract

Synthesis of silver nanoparticles using extracts from plants is an advantageous technological alternative to the traditional colloidal synthesis due to its simplicity, low cost, and the inclusion of environmentally friendly processes to obtain a new generation of antimicrobial compounds. The work describes the production of silver and iron nanoparticles using sphagnum extract as well as traditional synthesis. Dynamic light scattering (DLS) and laser doppler velocimetry methods, UV-visible spectroscopy, transmission electron microscopy (TEM) combined with energy dispersive X-ray spectroscopy (EDS), atomic force microscopy (AFM), dark-field hyperspectral microscopy, and Fourier-transform infrared spectroscopy (FT-IR) were used to study the structure and properties of synthesized nanoparticles. Our studies demonstrated a high antibacterial activity of the obtained nanoparticles, including the formation of biofilms. Nanoparticles synthesized using sphagnum moss extracts likely have high potential for further research.

## 1. Introduction

The synthesis of nanoparticles using biological organisms has received increasing attention in the last decade due to the growing need to develop safe technologies for the synthesis of nanomaterials. Only in recent years has the method of metal nanoparticle synthesis using plant extracts arisen as an alternative to chemical and physical methods [1,2]. The high interest in the synthesis of metal nanoparticles is related to their inhibitory effect against bacteria and fungi [3,4].

The standard synthesis of metal nanoparticles is mostly performed by physical or chemical methods [5,6]. Physical methods require costly equipment, while chemical methods consume expensive and toxic chemicals that are environmentally hazardous. In contrast, nanoparticles obtained with microorganisms are easy to synthesize and they have demonstrated biocompatibility and efficacy in therapeutic applications against bacterial infections and cancer treatments [7]. Furthermore, such particles stabilized with extracts are safer than conventional chemical ones, as toxic chemicals can remain in synthesized nanoparticles and limit their use in food industries, medicine, and agriculture. To mitigate the problems of expensive equipment and toxic chemicals, numerous methods for nanoparticles synthesis have been developed that use biological organisms [8,9,10,11].

Currently, the use of biological molecules as matrices is increasing, and plants, plant waste, bacteria and fungi are often used for nanoparticle synthesis [10]. Microorganisms such as bacteria, fungi and small algae can be used as templates for obtaining complexly ordered assemblies of in situ synthesized metal nanoparticles [12]. Thus, using *Chlorella pyrenoidosa* cells as biotemplates for the synthesis of iron nanoparticles, tiny magnetic-field-driven microrobots capable of targeted drug delivery were created [13]. Plant extracts are preferred over other biological sources because of their ample availability and wide range of reducing metabolites. The reducing properties of these antioxidant metabolites suggest the higher potential ability of plant extracts to synthesize nanoparticles with improved characteristics [14]. The rate of plant-mediated biological synthesis is higher than that of microorganism-based methods, and the nanomaterials obtained are more stable and diverse in shape and size. The presumed advantages include a speed of synthesis, environmental friendliness, and cost-effectiveness for the mass production of nanomaterials [9,10]. During that synthesis, extracted phytochemicals added to solutions of metal salts reduce metal ions and attach to the synthesized NPs, acting as stabilizing agents that allow better control of nanoparticle crystal growth [11,15,16].

In our work, a representative of the lower plants, *S. fallax* moss, was chosen as a plant component. Sphagnum mosses are common inhabitants of upland and transitional bogs. The ability of Sphagnum to inhibit decay processes is due to the high content of natural antibiotics, which are considered one of the most popular antiseptics in medicine. The metabolite profile of *S. fallax* consists mainly of acid-like and flavonoid glycoside compounds, which can act as powerful antimicrobial compounds, allowing the plant to control its environment. A comparison of the metabolite composition of *S. fallax* with previously known antimicrobial plant metabolites confirmed the presence of seventeen antimicrobial compounds, most of which were acids and glycosides [17].

In this study, Ag and Fe metal-based nanoparticles were either synthesized with sphagnum extracts or by classical chemical methods. In recent years, Ag nanoparticles have attracted attention due to their distinctive properties such as high electrical conductivity, chemical stability, near-infrared absorption and efficient charge separation [18]. They can be used as preservatives, effective antimicrobial and anticancer agents, and biomedical sensors and detectors that exhibit low toxicity for in vitro and in vivo applications [14]. Environmentally benign procedures of iron nanoparticles synthesis are also advantageous as they can reduce production of toxic elements [6,19].

The aim of the present study was to compare the characteristics of silver and iron nanoparticles obtained using different reducing agents. As far as we know, there are few studies that comprehensively compare nanoparticles obtained by different methods. We believe that such studies are highly relevant for selecting the most appropriate method of synthesis in the future.

## 2. Results and Discussion

### 2.1. Characterization of Metal Particles

#### 2.1.1. Synthesis of Metal Nanoparticles

The addition of *S. fallax* extract to aqueous solutions of AgNO_3_ and FeCl_3_ resulted in a color change of the mixtures from transparent to colloidal brown or gray, indicating the formation of Ag and Fe nanoparticles (Figure S1). Ag and Fe nanoparticles were also obtained by classical synthesis methods. The reduction of AgNO_3_ in an aqueous solution is one of the most widely used methods for the synthesis of Ag nanoparticles. According to previously published data [11] a color change is an important indicator of the synthesis of AgNPs. Ag nanoparticles appear brown in an aqueous environment as a result of surface plasmon oscillations. Other studies have reported similar color changes to dark brown that confirmed the formation of Ag and Fe nanoparticles [11,20,21,22].

#### 2.1.2. UV-Visible Absorption Spectroscopy

**Figure 1:**
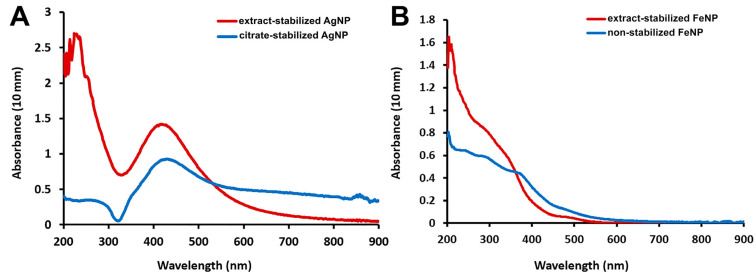
Absorption spectra of AgNPs (**A**) and FeNPs (**B**).

UV-visible absorption spectroscopy is the most widely used method for the preliminary analysis of metal nanoparticle formation [23]. Measurements in the UV-visible wavelength range showed that extract-stabilized Ag nanoparticles showed an absorbance peak at 420 nm (Figure 1A), which was in agreement with previously published results [10,11,24]. The maximum absorption of citrate-stabilized AgNPs was observed at 425 nm. The optical absorption spectra of noble metal nanoparticles and their intense color in water result from the phenomenon of surface plasmon resonance. Various parameters such as temperature, pH, concentration, size and shape of the synthesized nanoparticles affect the plasmon resonance oscillations [25]. The peak position and sharpness change in proportion to the size and shape of the nanoparticles and shift towards longer wavelengths with increasing particle size [24]. Thus, the close peak positions of extract-stabilized AgNPs and citrate-stabilized AgNPs suggest that AgNPs of similar sizes were obtained by both methods.

The differences in the absorbance peak location were more significant for FeNPs: the absorption of extract-stabilized FeNPs peaked at 272 nm, while that of non-stabilized FeNPs peaked at 250 nm. For extract-stabilized FeNPs, the peak in the same region was observed earlier in other studies [11,26]. UV-visible spectroscopy demonstrated the successful formation of Ag and Fe nanoparticles by the synthesis methods.

#### 2.1.3. Particle Size and Stability

**Figure 2:**
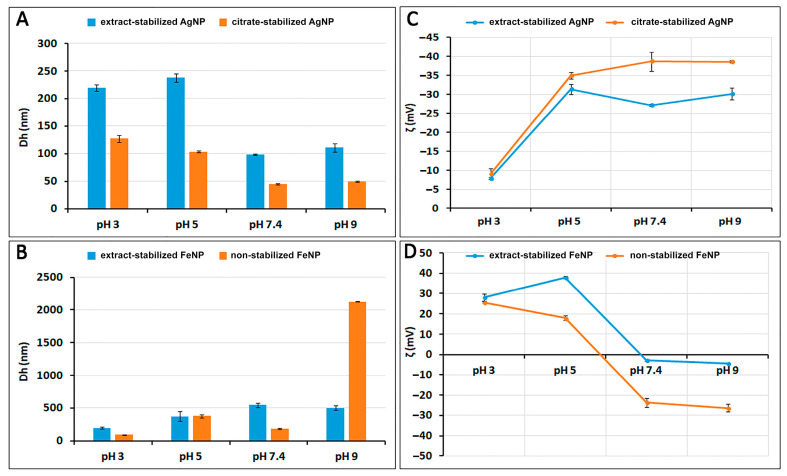
Hydrodynamic diameters (Dh) of AgNPs and FeNPs (**A**,**B**) and zeta potentials (ζ) of AgNPs and FeNPs (**C**,**D**) at different pH values.

The crucial characteristics of nanoparticles are their size and shape, as these parameters can affect other properties such as colloidal stability [27]. To characterize particle size and charge at different pH values, the hydrodynamic diameters (Dh) and zeta potentials (ζ) of nanoparticles were determined by dynamic light scattering (DLS) and laser Doppler velocimetry (Figure 2). The pH of the medium is important as it can influence the surface plasmon resonance oscillations and change the size, surface charge, and stability of the synthesized nanoparticles [25].

The repulsive force between suspended nanoparticles is caused by the zeta potential, which changes as the surface charge of the particles increases. The zeta potential is known to be an indicator of dispersion stability [28]. The zeta potential of any dispersion is affected by surface chemistry as well as pH. Figure 2D shows that when the pH of FeNP suspensions is more than 7, the surface of the particles becomes negatively charged. The surface of the particles has a positive charge when the pH of the suspension decreases below 6. The highest value of the zeta potential is observed in acidic and weakly acidic environments. At pH 3, ζ is 28.1 mV for extract-stabilized FeNPs and 25.7 mV for non-stabilized FeNPs. At higher zeta potential values, the particle size decreases from 370 (pH 5) to 198.3 nm for extract-stabilized FeNPs and 90.9 nm for non-stabilized FeNPs (pH 3). In contrast, at pH 9, ζ is −4.26 mV for extract-stabilized FeNPs and −26.3 mV for non-stabilized FeNPs, while the particle size increases to 502.2 nm for extract-stabilized FeNPs and 2125 nm for non-stabilized FeNPs (Figure 2B). AgNPs have the highest zeta potential value in weakly acidic and alkaline environments. Figure 2C shows that at pH 3, the charge decreases from −35 (pH 5) to −7.91 for extract-stabilized AgNPs and −9.09 for citrate-stabilized AgNPs.

Thus, suspensions of AgNPs and FeNPs possess electrostatic stability due to the strong repulsive force between the charged particles. At lower pH for FeNPs and higher pH for AgNPs, the particles have high surface charge and high zeta potential values, preventing particle collision and aggregation.

**Figure 3:**
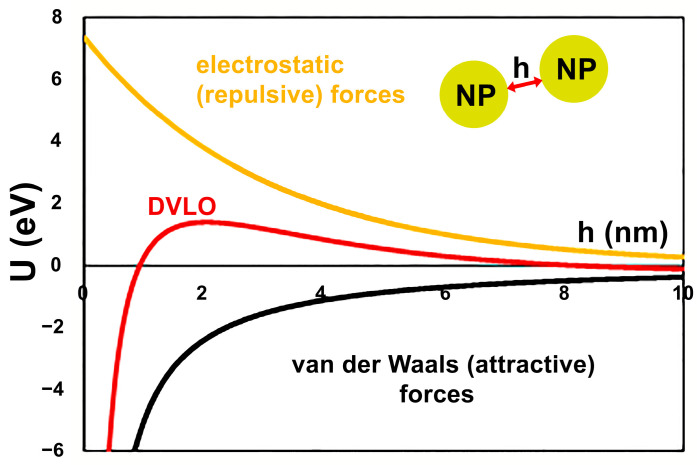
Schematic diagram of evaluation on colloidal stability of extract-stabilized FeNPs.

In order to analyze the cumulative effect of the NPs size and *ζ*-potential on their colloidal stability, the energy of inter-particle interactions has been considered in the view of Derjaguin, Landau, Verwey and Overbeek (DLVO) theory. The van der Waals potential has been assessed using classical approach developed by Hamaker [29] for the case of two identical spherical particles (Equation (1)):

(1) $$U_{vdW}=-\frac{A_{H}}{6}\cdot \left[\frac{d^{2}}{2h\left(h+2d\right)}+\frac{d^{2}}{2{\left(h+d\right)}^{2}}+\ln\left(\frac{h\left(h+2d\right)}{{\left(h+d\right)}^{2}}\right)\right]$$

where *A_H_* is the Hamaker constant for the NP in given medium, *d* is the diameter of NP, *h* is the surface-to-surface separation distance, as illustrated in Figure 3.

For the calculations, Hamaker constants of 6.16 × 10^−20^ J and 5.38 × 10^−20^ J have been taken for AgNPs and FeNPs [30] in water, respectively. It is of note that the values of the Hamaker constants vary in the literature, which may result in the inaccuracy of the calculations. However, if so, this inaccuracy would bring systematic error to all the calculations and therefore the results obtained for the same materials (Ag or Fe) can be compared. Average diameters presented in Figure 2 have been used for the calculations.

In order to evaluate the electrostatic repulsion between the NPs, the energy of electrostatic interactions between two spherical colloids of the same charge has been estimated in accordance with the Equation (2) [31]:

(2) Uels≈πεε0dζ2·e−hλD

where *ɛ* and *ɛ*_0_ are the dielectric constants of the medium (taken as 78.4 for the water) and vacuum (8.85·10^−12^ F⋅m^−1^), respectively, *ζ* is *zeta*-potential of the NPs as measured by the DLS (Figure 2), and $\lambda_{D}$ is the Debye length. In Equation (3):

(3) $$\lambda_{D}=\sqrt{\frac{\varepsilon \varepsilon_{0}k_{B}T}{2N_{A}Ie^{2}}}$$

*k_B_* is the Boltzmann constant (1.38·10^−23^ J⋅K^−1^), *T* is the absolute temperature (293 K), *I* is the ionic strength of the medium (0.01 M), *N_A_* is the Avogadro number, and *e* is the elementary charge.

Notably, Equation (2) is valid for hλD >> 1.

As illustrated in Figure 3 for the case of as prepared FeNPs, the DVLO potential of the interaction between the NPs is assessed as the sum of van der Waals and electrostatic potentials. The estimated DVLO profile is typical for the colloidal suspension of identical particles, wherein the van der Waals forces dictate the profiles at small distances, while the double layer force dominates the intermediate distances.

**Figure 4:**
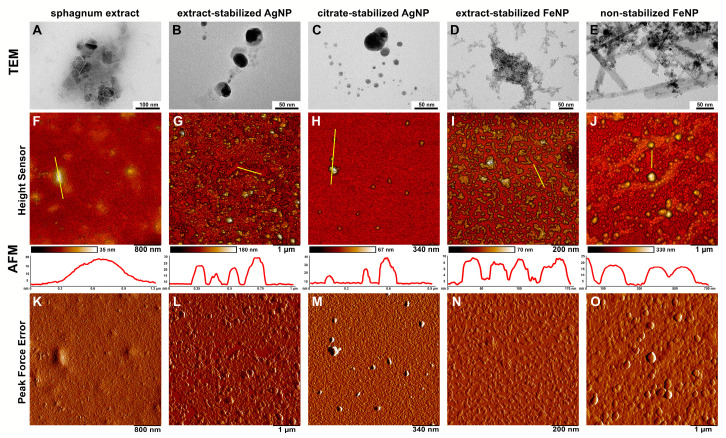
TEM and AFM microscopy of AgNPs and FeNPs. (**A**–**E**)—TEM images; (**F**–**O**)—AFM images; (**F**–**J**)—topography images (Height Sensor channel), with yellow bars marking the areas corresponding to the topography plots shown below; (**K**–**O**)—topography images (Peak Force Error channel); (**A**,**F**,**K**)—sphagnum extract; (**B**,**G**,**L**)—extract-stabilized AgNPs; (**C**,**H**,**M**)—citrate-stabilized AgNPs; (**D**,**I**,**N**)—extract-stabilized FeNPs, (**E**,**J**,**O**)—FeNPs.

The size and morphology of the obtained particles were also investigated by TEM and AFM (Figure 4). The magnification factor in TEM is usually in the range of about 10^2^–10^6^, allowing detailed observations of the nanoparticle structure [32]. AFM is also an excellent method for determining the size and morphology of particles [6]. Using TEM and AFM, the morphology of the synthesized nanoparticles was determined, as well as their size, size distribution, shape, shape heterogeneity and aggregation.

TEM and AFM micrographs of Ag nanoparticles obtained with different methods demonstrate particles of uniform spherical shape of up to 100 nm in diameter, which can aggregate into agglomerates, especially in citrate-stabilized variant (Figure 4). These agglomerates are easily dispersed in water by ultrasonic treatment. The results are in good agreement with previously published data, where Ag nanoparticles of different sizes (from 5 to 100 nm) and morphologies (spherical, nanorods, hexagonal, etc.) could be obtained using various plant extracts such as soluble starch, coffee, green tea, mushrooms, spices and others [10,33,34]. The measured sizes of Fe nanoparticles differed depending on the way of synthesis (Figure 4). The non-stabilized FeNPs were spherical in shape and up to 120 nm in size, and were highly aggregated, whereas the extract-stabilized FeNPs were lamellar in shape with up to 50 nm in diameter and up to 20 nm in thickness, and weakly aggregated. The similarly shaped FeNPs were previously obtained using strawberry *Fragaria ananassa* leaves [11].

**Figure 5:**
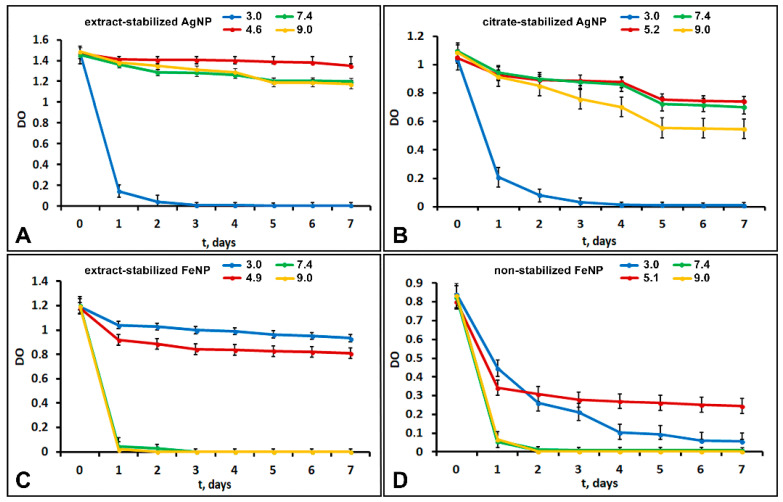
The dependence of the optical density of suspensions of extract-stabilized AgNPs (**A**) and citrate-stabilized AgNPs (**B**) and extract-stabilized FeNPs (**C**) and non-stabilized FeNPs (**D**) on time at different pH values.

The size and morphology of nanoparticles strongly affect their colloidal stability. The stability of aqueous suspensions of metal nanoparticles was evaluated by the sedimentation rate. Figure 5 shows the dependences of the optical density of nanoparticle suspensions on time at different pH values. Figure 5 shows that the stability of silver nanoparticle suspensions at pH 3 drastically decreased within 3 days, with a corresponding decrease in the optical density. The particles precipitated almost completely within two days. This low stability was probably related to changes in the particle ξ-potentials at pH 3, which decreased from −31 to −7.9 mV for extract-stabilized AgNP and from −35 to −9 mV for citrate-stabilized AgNP. When the potential is too low the particles collide and aggregate due to attractive intermolecular forces, and then the aggregated particles precipitate.

In slightly acidic (pH 4.6–5.2), neutral (7.4) and slightly alkaline (9.0) environments, extract-stabilized AgNP showed good sedimentation stability with a minor decrease in the optical density of the suspension over seven days. For extract-stabilized AgNPs, the state of dynamic equilibrium was reached in the time period from 2 to 7 days. Citrate-stabilized AgNPs showed lower sedimentation stability under the same conditions. Dynamic equilibrium was reached between days 5 and 7, so citrate-stabilized AgNPs were less stable than extract-stabilized ones.

Extract-stabilized FeNPs particles at pH 3.0 and 4.9 showed good sedimentation stability. A state of dynamic equilibrium was reached between days 2 and 7. The particle size decreased from 375 at pH 4.9 to 198 nm at pH 3. Non-stabilized FeNPs in acidic and weakly acidic environments showed low sedimentation stability; the optical density of FeNPs suspension at pH 3.0 gradually decreased, and the particles almost completely settled within 7 days. The optical density of the FeNPs suspension (pH 5.1) decreased over 24 h; the state of dynamic equilibrium was reached from 3 to 7 days. Thus, at acidic pH, extract-stabilized FeNPs were more stable than non-stabilized ones.

The increase in the medium pH to 7.4 and 9.0 was accompanied by a drastic decrease in the optical density of extract-stabilized FeNP and non-stabilized FeNP suspensions. The particles were completely settled within 2 days. For extract-stabilized FeNP, the reason could be the decrease in the ξ ζ -potential from 37 to −4 mV leading to particle aggregation and an increase in their size from 375 to 500 nm followed by the sedimentation of FeNP aggregates.

**Figure 6:**
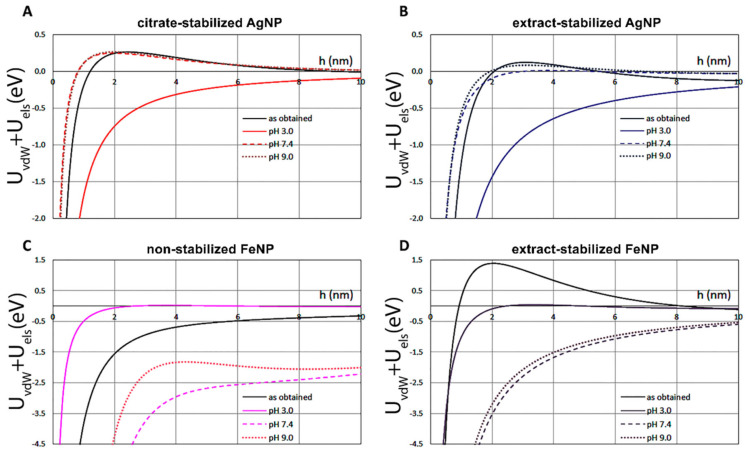
Evaluation of the influences of pH and synthesis method on the energy profiles of AgNPs (**A**,**B**) and FeNPs (**C**,**D**).

The effect of pH and the method of NP fabrication on the energy profiles are illustrated in Figure 6 for both AgNPs (A,B) and FeNPs (C,D). The repulsion was slightly higher for citrate-stabilized AgNPs in the comparison to extract-stabilized AgNPs, suggesting their better colloidal stability.

The DVLO profiles also predict that AgNPs are more stable at elevated pH, and this trend is quite similar for both extract-stabilized AgNPs and citrate-stabilized AgNPs. In the case of FeNPs, no clear dependence of the potential on the pH is observed: the repulsion significantly increases at acidic pH 3.0 and is also higher at pH 9.0 than that at pH 7.4, suggesting the minimal stability of both non-stabilized FeNPs and extract-stabilized FeNPs at neutral pH. Interestingly, the free energy of attraction of as-obtained extract-stabilized FeNP is the highest among all considered cases.

Overall, the results showed that extract-stabilized AgNPs and FeNPs are more stable. Good sedimentation stability is observed within pH 4.5–9 for Ag nanoparticles and pH 3–5.5 for Fe nanoparticles.

#### 2.1.4. Energy Dispersive X-ray Spectroscopy (EDS) Analysis

EDS is a reliable method that allows elemental analysis of the surface of solid samples, such as synthesized nanoparticles. EDX microanalysis is performed by measuring the energy distribution and intensity of X-ray signals generated by an electron beam focused on the sample [35]. EDS spectra taken from AgNPs and FeNPs confirmed the synthesis of metal particles by a sphagnum extract and the classical synthesis method (Figure S2). According to previous reports, AgNPs typically absorb energy in the 3 keV region due to surface plasmon resonance. Other weak peaks observed in the EDS spectra could be caused by biomolecules bound to the AgNP surface [21] FeNPs absorb energy in the 6–7 keV region [11].

Figure S2A shows the EDS spectrum of the sphagnum extract, which shows numerous peaks that are indicative of a complex chemical composition, but no metallic Ag and Fe peaks were detected. Additional STEM imaging and elemental mapping (Figure S3) demonstrated the presence of Cl impurities in the extract-stabilized AgNP sample, probably derived from the Sphagnum extract [11]. Furthermore, as can be seen in Figure S2 and Figure S3, metal positions coincide with the non-metallic elements, indicating that they are in close contact.

#### 2.1.5. Dark-Field Microscopy with Hyperspectral Analysis

A specific property of extract-stabilized silver nanoparticles was found during microscopic observations, which was a “maturation” of nanoparticles under the influence of light. Dark-field photographs of extract-stabilized silver nanoparticles taken at different time intervals, 0, 10 and 20 min, demonstrate the change in the spectral properties of the particles over time (Figure S4, Video S1). Blue light was previously shown to promote the synthesis of extract-stabilized silver nanoparticles, probably by reduction of metal ions mediated by serine/threonine protein kinase of the plant extract [36]. However, as we boiled a moss extract for 30 min, all the proteins most probably became denatured during the boiling process and the activity of all enzymes was lost.

Hyperspectral analysis has shown that the spectral properties of silver nanoparticles depended on the apparent particle color. Thus, the peak of yellow silver particles was located in the yellow spectral region of 570–590 nm, the peak of the blue particles was shifted to the blue range of 525–575 nm, the green particles had a peak in the 510–550 nm region, and the red ones in the 630–780 nm region (Figure S5). It is known that the color of metal nanoparticles depends on their shape and size [37]. Thus, the hyperspectral profile points not only to the color, but also to the size of the silver particles.

In further analysis, when comparing the spectral properties of different metal particles, the averaged spectral signals were used, since FeNPs do not demonstrate such color-dependent spectral profiles. In averaged spectra, iron nanoparticles had a shoulder in the region of 500–575 nm, and silver nanoparticles had a broad peak of 500–600 nm.

**Figure 7:**
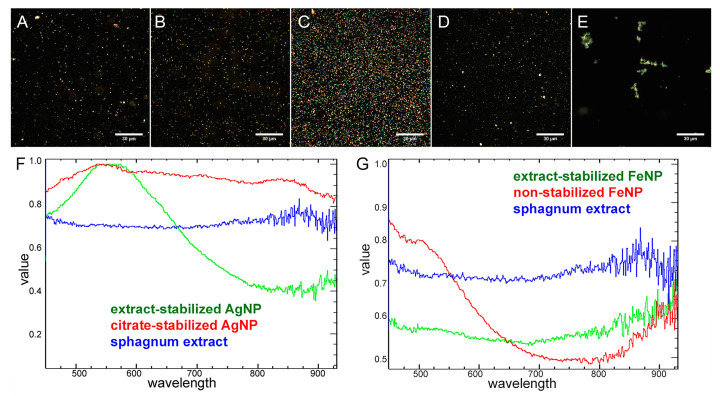
Dark-field photographs (**A**–**E**) and normalized reflected light spectra (**F**,**G**) of sphagnum extract (**A**), extract-stabilized (**B**) and citrate-stabilized (**C**) silver particles, extract-stabilized (**D**) and non-stabilized (**E**) iron particles. Spectral data were obtained for 50 particles of different colors.

Hyperspectral characterization of silver nanoparticles stabilized with different polymers was performed earlier in the works of our laboratory [5]. The spectral profiles of silver nanoparticles stabilized with different polymers differed significantly, which allowed identification of the type of coating based on the spectra [5]. The stabilization of metal nanoparticles with a sphagnum extract also revealed itself in the spectra. The spectral signal of sphagnum had a characteristic profile different from the spectra of synthesized particles, although pronounced peaks were absent. Application of the extract as a stabilizer led to a change in the spectral signals of the particles. There was a noise at the end of the spectra of sphagnum and nanoparticles synthesized using sphagnum extract, in the region of 800–930 nm (Figure 7). For particles obtained without sphagnum extract in this area, the plot was smoother and no noise was observed.

#### 2.1.6. FT-IR

**Figure 8:**
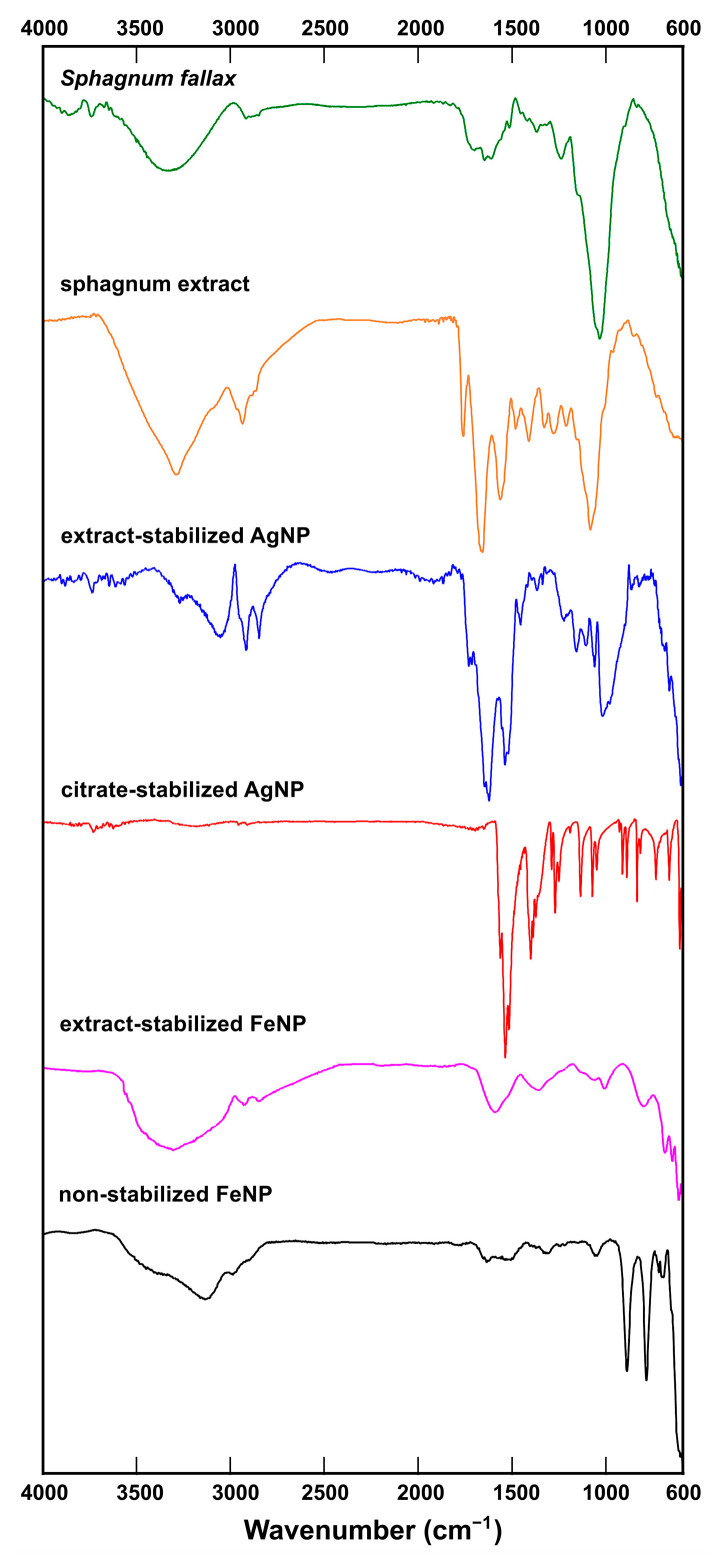
FT-IR spectra of a sphagnum leaf, sphagnum extract, extract-stabilized AgNPs, citrate-stabilized AgNPs, extract-stabilized FeNP, and non-stabilized FeNP (top to bottom).

To study the composition of Ag and Fe nanoparticles, FT-IR spectrometry was carried out. FT-IR spectra were recorded in the range of 600–4000 cm^−1^ to recognize functional organic groups or biomolecule residues (Figure 8). In the IR-spectrum of a *S. fallax* leaf, bands associated with the presence of alcohols or carbohydrates were found (O-H stretching vibrations at 3336 cm^−1^ and the C-O stretching vibrations at 1237 cm^−1^ and 1035 cm^−1^). Apparently the *S. fallax* leaf spectrum was dominated by cellulose. The presence of amides was indicated by a band at 1515 cm^−1^ related to NH out-of-plane bending and another around 1646 cm^−1^ belonging to C=O stretching vibrations in amides.

In Sphagnum extract, the typical bands for alkanes were observed as C-H stretching vibrations near 2925 cm^−1^ and C-H rocking vibrations at 1383 cm^−1^ with C–H bending or scissoring at 1451 cm^−1^. C-O stretching vibrations from 970 cm^−1^ to 1250 cm^−1^ typical of alcohols and carbohydrates were also found. A strong, somewhat broad band around 3280 cm^−1^ probably belonged to the N-H stretching vibration in amides, which also showed a C=O stretching band in the middle of the spectrum at 1632 cm^−1^ and a band at 1536 cm^−1^ corresponding to NH out-of-plane bending. A band at 1737 cm^−1^ was probably related to C=O stretching. Thus, according to the IR-spectrum, the Sphagnum extract was enriched with carbohydrates (or alcohols), and amides.

The prominent band in the spectrum of extract-stabilized AgNPs was that at 3054 cm^−1^ corresponding to the N–H stretching vibrations. The NH2 scissoring vibrations in amides could also reveal themselves as the band at 1623 cm^−1^.The bands at 1731 cm^−1^ and 1716 cm^−1^ could be ascribed to C=O stretching vibrations. The band at 1538 cm^−1^ could indicate NH out of plane bending in amides together with a small band at 1646 cm^−1^ corresponding to C=O stretching vibrations in amides. The bands typical to alkanes were observed as C-H stretching vibrations near 2918 cm^−1^ and 2848 cm^−1^.

The FT-IR spectrum of citrate-stabilized AgNPs confirmed the successful stabilization of the nanoparticles with citrate, as the spectrum was very close to that of citric acid published elsewhere [38], including the peaks at 1271 cm^−1^ and 1400 cm^−1^ corresponding to C-O stretching vibrations in carboxylic acids.

In the spectrum of non-stabilized FeNPs, the most prominent band was that at 3136 cm^−1^ corresponding to O–H stretching vibrations.

The spectrum of extract-stabilized FeNPs showed bands related to alkanes (at 2919 cm^−1^ and 2850 cm^−1^ due to C-H stretching vibrations) and amides (band at 1371 cm^−1^ due to C-N stretching vibrations). In addition, C=O stretching vibrations at 1000-1200 cm^−1^ and O-H stretching vibrations between 3500–3200 cm^−1^, typical of alcohols, were observed. 

In Figure 8 one can see that the similar absorption bands at 2929, 2850, 1737, 1630, 1540, 1455, 1380 cm^−1^ and a band at 1030–1060 cm^−1^ could be traced in the spectra obtained from sphagnum leaves, sphagnum extract and extract-stabilized AgNPs and FeNPs. The obtained FTIR spectra confirmed the interaction of sphagnum biomolecules with synthesized Ag and Fe nanoparticles.

### 2.2. Toxicity Assessment of Metal Nanoparticles

#### 2.2.1. Suppression of Biofilm Growth

Biofilm growth is inherent to most known bacteria [39]. In the process of biofilm formation, bacteria attach to solid surfaces, then multiply to form microcolonies and synthesize extracellular polymeric substances, forming a matrix that gives structure to the biofilm. Biofilm growth is often triggered by stressful environmental conditions. It is tightly regulated in response to environmental stimuli and is highly species and strain dependent. Bacteria in biofilms are more resistant to chemical and physical impacts [40] and antibiotics [41] than planktonic bacteria and thus pose a clinical challenge in healthcare facilities [42]. Thus, the search for agents that would inhibit biofilm growth is an important area of research. Extract-stabilized nanoparticles, especially AgNPs, are considered very promising in the fight against microbial biofilms.

**Figure 9:**
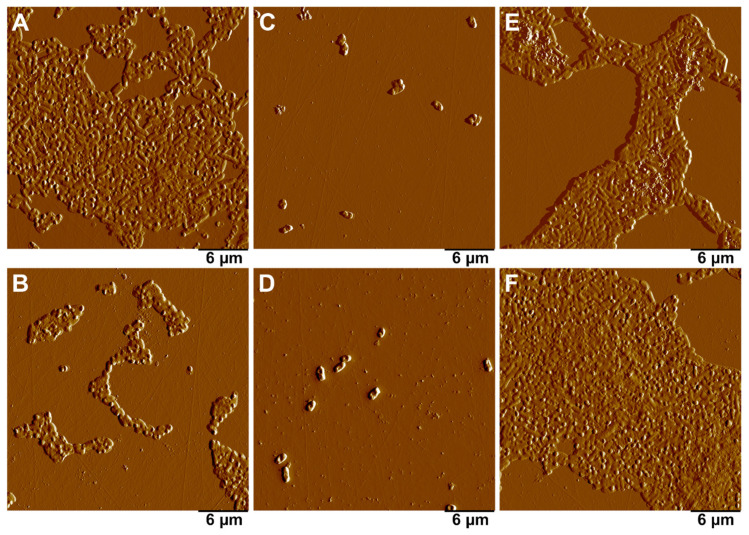
AFM images of the biofilms formed by *E.coli* strain MG1655 after 24 h co-culture with silver and iron nanoparticles. (**A**)—control; (**B**)—10% sphagnum extract; (**C**)—citrate-stabilized Ag nanoparticles; (**D**)—extract-stabilized Ag particles; (**E**)—non-stabilized Fe particles; (**F**)—extract-stabilized Fe particles.

The obtained nanoparticles were analyzed for their effectiveness in suppressing the growth of biofilms of *E. coli* strain MG1655 when co-cultured for 24 h. The obtained biofilms were visualized using atomic force microscopy (Figure 9). Prior to the testing, in order to assess the microbiological purity of the sphagnum extract and silver and iron nanoparticles, their inoculation on a solid agarized nutrient medium was performed. This assessment showed the absence of bacteria in the samples (Figure S6).

Both extract-stabilized and citrate-stabilized AgNPs showed strong antibacterial activity, completely suppressing the growth of *E. coli* strain MG1655 biofilms (Figure 9C,D). In the presence of AgNPs, single bacterial cells were preserved, but the biofilm was not formed. It is worth noting that the sphagnum extract itself led to a delay in biofilm growth (Figure 9B), while FeNPs were inefficient in their inhibition of biofilm formation (Figure 9E,F). A conclusion can be proposed that the inhibition of biofilm formation was mostly due to the action of AgNPs, and coating with sphagnum biomolecules did not play a significant role in suppressing biofilm formation by *E. coli* strain MG1655 bacteria.

#### 2.2.2. Cytotoxicity on Human Cell Cultures

**Figure 10:**
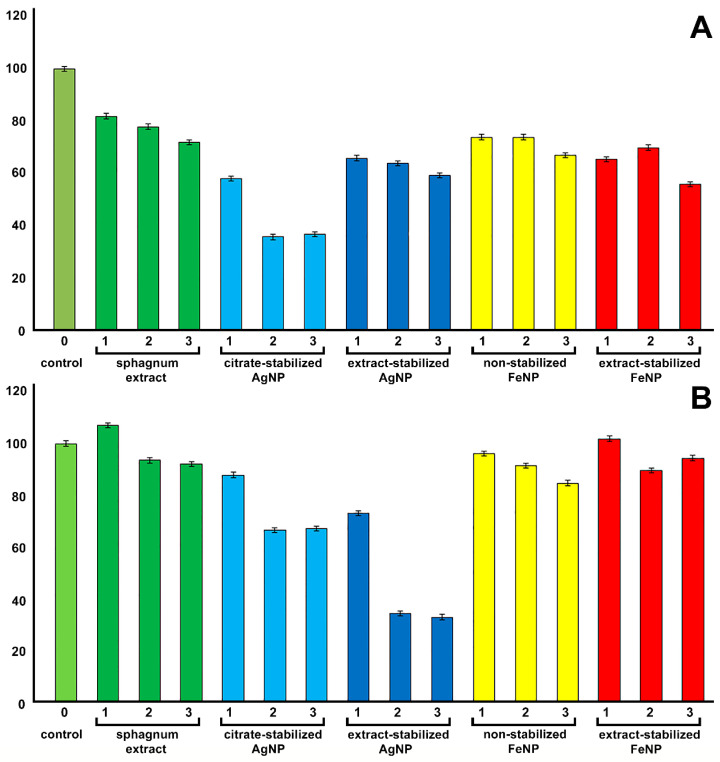
Assessment of the viability of A549 cells (**A**) and hMSCs (**B**) after 24 h of co-incubation with nanoparticles in various concentrations (1—1%, 2—5%, 3—15% for sphagnum extract; 1—1 µg/mL, 2—5 µg/mL 3—15 µg/mL for Ag and Fe particles).

Two cell lines (Figure 10) (human lung carcinoma cells (A549) and human mesenchymal stem cells (MSCs)) were used to determine the cytotoxic effect of the synthesized nanoparticles, and a standard MTT test was applied [6]. It is known that MTT is reduced by cytosolic enzymes of viable cells to purple formazan and the effect of nanoparticles on cells can be determined by the change in optical density in the treated variants relative to the control.

It was shown that the sphagnum extract in concentrations of 1, 5 and 15% had no toxic effect on the metabolic activity of hMSCs, while the concentration of sphagnum extract in the medium of 15% reduced the viability of A549 cells by 20% relative to controls. The introduction of citrate-stabilized silver nanoparticles into the medium at concentrations above 5 μg/mL reduced the viability of A549 cells by 60%, while extract-stabilized silver nanoparticles were less toxic to A549 cells. However, silver nanoparticles obtained with sodium citrate were less toxic for hMSC cells, reducing cell viability by only 30% relative to the control at the concentration of 15 µg/mL. On the contrary, the introduction of extract-stabilized silver nanoparticles to the incubation medium at a concentration higher than 5 µg/mL reduced cell viability by almost 70% relative to the control.

The iron nanoparticles obtained by different methods were non-toxic for both cell types. The non-toxicity of non-stabilized magnetic nanoparticles to A549 cells was previously shown [6]. Stabilization of iron nanoparticles with different polyelectrolytes, e.g., PEI, resulted in strong toxic effects [6]. The production of stable and non-toxic iron nanoparticles on the basis of *S. fallax* extract opens up opportunities for the use of bio-stabilized FeNPs in cell biology as drug delivery agents or as safe components of the substrates for the differentiation of stem cells [43].

The effect of silver nanoparticles on cells depends on the synthesis process, the exposure time, concentration, temperature, particle size, coating and cell line used [44]. The 50% lethal dose (LD50) of silver nanoparticles varies for different organisms and cell lines. For example, for embryonic zebrafish, it was found to be 125 μg/mL [45]. The LC50 of tea-based nanoparticles for HT-29 human colon cancer cell line was 71.5 µg/mL, whereas for MCF-7 human breast cancer cell line it was 59.2 µg/mL and for normal human lung fibroblast cells (IMR-90) the LC50 of starch-coated silver nanoparticles it was 50 µg/mL [46]. In HepG2 cells, a concentration of 12.5 µg/mL of silver nanoparticles coated with glycolipids caused death in 42% of cells [47]. At the same time, in some works, it was found that the LC50 for A549 cells of extract-stabilized silver nanoparticles obtained using different organisms was 50 µg/mL [48,49].

Silver nanoparticles obtained with biological reducing agents were previously reported to be less toxic than silver nanoparticles obtained with citrate [50]. One of the reasons for the higher toxicity of extract-stabilized silver nanoparticles found in our study was probably their small size, about 20–100 nm. Thus, Li et al. showed that silver nanoparticles of 60–70 nm had negligible cytotoxicity even at a relatively high dose of 125 µg/mL, while silver nanoparticles with a size of 25 to 45 nm at the same concentration were toxic, leading to the death of more than 50% of cells [51]. Cell death under AgNP treatment could be associated with oxidative stress of cells due to a decrease in glutathione (GSH) and superoxide dismutase (SOD) levels and an increase in lipid peroxidation, which eventually leads to apoptosis by increasing caspase-3 activity and DNA fragmentation [52].

## 3. Materials and Methods

### 3.1. Particle Synthesis

#### 3.1.1. Preparation of the Extract

The *S. fallax* extract was prepared by a method similar to that described in [11]. Sphagnum was first ground to powder in a porcelain mortar. Next, 5 g of dried sphagnum powder was mixed with 100 mL of distilled water and boiled at 100 °C for 30 min with constant stirring on a magnetic stirrer at 600 rpm. Then, the resulting mixture was completely cooled for 12 h to room temperature. The resulting extract was purified of sphagnum particles by centrifugation at 3500 rpm for 20 min, the supernatant was collected, and filtered through filter paper. As a result, a 5% solution of sphagnum extract was obtained.

#### 3.1.2. Extract-Stabilized Particle Synthesis

**AgNP.** Silver nitrate (AgNO_3_) was used as a precursor to produce silver nanoparticles. Extract-stabilized silver nanoparticles were obtained by reducing silver ions using sphagnum extract. The sphagnum extract acted as a stabilizer. A sample of 100 mL of 0.01 M AgNO_3_ solution was subjected to continuous stirring, heated to 90 °C and then 10 mL of 5% sphagnum extract was added drop by drop. After adding the entire extract, the resulting mixture was kept for 2 h under continuous stirring at 90 °C. After 60 min following addition of the extract, the reaction mixture changed to dark brown and became opaque, indicating the formation of silver nanoparticles. The resulting solution was left for 12 h at room temperature until completely cooled, and then for 24 h to mature. The resulting particles were precipitated by centrifugation at 13,000 rpm for 60 min and washed 3 times with distilled water. The precipitate was broken each time by ultrasound for better washing.

**FeNP.** Iron (III) chloride salt (FeCl_3_) was used for the synthesis of iron nanoparticles. 10 mL of sphagnum extract was added drop wise to 100 mL of a stirred 0.012 M FeCl_3_ solution. The resulting mixture was subjected to continuous stirring and heated to 90 °C. After adding the entire extract, the resulting mixture was kept for 2 h under continuous stirring at 90 °C. Thirty minutes after the addition of the extract, the reaction mixture changed from yellow to dark red, indicating the formation of iron nanoparticles. The resulting solution was left for 12 h at room temperature until completely cooled, and then for 24 h to mature. The obtained particles were precipitated by centrifugation at 13,000 rpm for 60 min at +4 °C and washed 3 times with distilled water. The precipitate was broken each time by ultrasound for better washing.

#### 3.1.3. Classical Synthesis Method of the Particles

**AgNP.** An aqueous solution (0.012 M, 100 mL) of AgNO_3_ was heated to 90 °C on a magnetic stirrer, and 10 mL of 5% sodium citrate was added dropwise while stirring. After adding all the sodium citrate, the mixture was kept for 2 h at continuous stirring at 90 °C. Ten minutes after the addition of sodium citrate, the reaction mixture changed from yellow to dark gray, indicating the formation of silver nanoparticles. The resulting solution was left for 12 h at room temperature until completely cooled, and then for 24 h to mature. The low molecular weight substances were removed by dialysis. The particles were collected by centrifugation at 13,000 rpm for 60 min.

**FeNP.** Magnetic nanoparticles were obtained by precipitation from solutions of Fe^2+^ and Fe^3+^ salts and NaOH solution [53]. Iron salts (2 M FeCl _2_ 4H_2_O and 1 M FeCl _3_ in 2M HCl) were mixed and heated to 75 °C. While stirring on a magnetic stirrer, 1.5 M NaOH was added. After a few minutes, the color of the solution changed from yellow to dark brown, indicating the formation of iron nanoparticles. After adding all components, the resulting mixture was kept for 2 h under continuous stirring at 75 °C. The resulting solution was left for 12 h at room temperature until completely cooled, and then for 24 h to mature. The resulting particles were precipitated by centrifugation at 13,000 rpm for 60 min and washed 3 times with distilled water. The precipitate was broken each time by ultrasound for better washing.

### 3.2. Particle Characterization

#### 3.2.1. Particle Size and Stability

The hydrodynamic diameter and zeta potential values of the nanoparticles were determined using the Zetasizer Nano ZS instrument, (Malvern Panalytical Ltd., Malvern, UK).

The stability of aqueous suspensions of metal nanoparticles was evaluated by the sedimentation rate of NPs. The sedimentation rates of the suspensions were determined spectrophotometrically (NanoPhotometer NP80, Implen, München, Germany) over 7 days at a concentration of 0.25 mg/mL. The measurements were performed in a 10 mm quartz cuvette at the following wavelengths, corresponding to the adsorption maximum of the suspensions: 420 nm for extract-stabilized AgNP, 425 nm for citrate-stabilized AgNP, 272 nm for extract-stabilized FeNP, and 350 nm for non-stabilized FeNP.

The stability of aqueous suspensions at different pH values was also evaluated. The required pH value (pH 3.0, 7.4, and 9.0 at 25 °C) of the solutions was set by adding hydrochloric acid or sodium hydroxide solutions (0.1 mol/L) and monitored using a SevenCompact S220 pH meter (Mettler Toledo, Schwerzenbach, Switzerland).

#### 3.2.2. Dark-Field Microscopy and Hyperspectral Particle Assessment

Dark-field images and reflected light spectra were obtained using an Olympus BX51 microscope (Olympus, Tokyo, Japan) equipped with a CytoViva^®^ enhanced dark-field condenser (CytoViva, Auburn, Al, USA) with a Fibre-Lite DC-950 halogen light source (150 W) (Dolan Jenner Industries Inc., Boxborough, MA, USA).

Dark-field images were acquired at exposure times of 154 ms and 575 ms using Exponent 7 software (Dage-MTI, Michigan City, IN, USA). Spectra were recorded using a Specim V10E spectrograph (Spectral Imaging LTD, Oulu, Finland) and a Pixelfy.usb CCD camera in the wavelength range from 400 to 1000 nm with a spectral resolution of 2 nm. HSI data were collected from 900 scan lines at 0.25 s exposure and full light intensity, which took about 11 min to obtain a full scan. The hyperspectral data were stored and analyzed as hypercubes using ENVI software v. 4.8 (Harris Geospatial Solutions, Broomfield, CO, USA) [54]. HSI data were corrected to eliminate the lamp spectrum. Spectral signals were either collected from 20 to 30 individual particles of a certain color (6000 pixels) or averaged from 50 particles of different colors (6000 pixels). As a result, hyperspectral libraries were obtained. To avoid sample contamination, ultrapure Nexterion^®^ slides and coverslips (Schott, Mainz, Germany) were used to obtain dark-field images and spectra.

#### 3.2.3. FT-IR

FT-IR was performed by a FT-IR spectrophotometer FT-801, equipped with ZnSe ATR prism (Simex, Novosibirsk, Russia). Spectra were recorded in the range of 600 to 4000 cm^−1^ at a resolution of 4 cm^−1^ and averaged from 26 scans. The samples were dried before FT-IR studies. The scans were processed using the Zair v 3.5 software (Simex, Novosibirsk, Russia).

#### 3.2.4. TEM and Energy Dispersive X-ray Spectroscopy (EDS) Analysis

The structure and morphology of the samples were studied using a transmission electron microscope (TEM) JEM-2100 (JEOL, Tokyo, Japan) with a magnification factor of 50–1,500,000 times and an image resolution of 0.19 nm at 200 kV. Before the study, each sample (10 µL) was dispersed in distilled water (0.5 mL). The resulting solution was applied to a copper grid. The sample holder was cleaned in a plasma chamber to avoid the ingression of organic compounds into the microscope. To calculate the average particle size, the obtained micrographs were processed using Image J software (V. 1.43).

Scanning transmission electron microscopy (STEM) was performed in combination with energy dispersive X-ray spectroscopy (EDX) to map the distribution of elements in the samples on the JEM-2100 instrument (JEOL, Tokyo, Japan). Sample preparation was carried out similarly to that for transmission electron microscopy.

#### 3.2.5. Atomic Force Microscopy

AFM images of nanoparticles and biofilms were obtained using a Dimension Icon microscope (Bruker, Billerica, MA, USA). The scans were performed in air at room temperature in PeakForce Tapping mode. Standard silicon nitride cantilevers ScanAsyst-Air (Bruker) were used (nominal length 115 μm, tip radius 2 nm, spring constant 0.4 Nm^−1^). Optimal scanning parameters were set to obtain high-quality topography images, while scanning force was 1–2 nN at a scanning speed of 0.8–0.9 Hz [55]. Images were acquired at a resolution of 512 scan lines. The maximum scan sizes for biofilms were 30 × 30 μm. Images were edited using Nanoscope Analysis v 1.7 software (Bruker, Billerica, MA, USA).

### 3.3. Toxicity Assessment

#### 3.3.1. Evaluation of the Microbiological Purity of Sphagnum Extract and Particles

Samples of sphagnum extract and obtained particles were seeded on the agarized nutrient broth medium and incubated at 37 °C for 24 h. The cultures were examined visually.

#### 3.3.2. Obtaining and Fixing Biofilms of *E. coli* Strain MG1655

Two mL of liquid NB medium, 200 μL of cell suspension prewashed in NaCl (0.9%), and 200 μL of extract/particles (1 mg/mL) were added to adhesive Petri dishes (3.5 cm). The cells were incubated for 24 h under agitation (300 rpm) in a plate incubator (Elmi ST-3, Riga, Latvia). Next, biofilm fixation was performed. One mL of 2.5% glutaric aldehyde was added to the Petri dish, incubated for 1 h, then thoroughly washed with distilled water 5 times with a 5 min incubation period. After fixation, biofilms were dried at room temperature for 24 h [56].

#### 3.3.3. Cell Viability of A549 and hMSC Cells

Cell viability after incubation with nanoparticles and nanoparticle precursors was assessed using the MTT test [57]. hTERT-transduced cells (hMSC) were obtained using lentiviral transduction. The pWPT-GFP construct (cat #12255, Addgene, Watertown, MA, USA) was applied to obtain hMSCs carrying the green fluorescent protein (GFP) gene. Then, cells with a high level of GFP expression were isolated using a FACS Aria III flow cytometer. Ninety-six clones were selected for each cell line. A549 (ATCC, Manassas, VA, USA) and hMSC cells were cultured in 96-well plates (Corning Inc., Corning, NY, USA) at a concentration of 3000 cells per well and treated with nanomaterials for 24 h at 37 °C. The cells were then incubated for 4 h at 37 °C with MTT solution (0.5 mg/mL) in PBS. Afterwards, the MTT solution was removed, and the precipitated dye was dissolved in DMSO and gently shaken for 15 min. Then, the change in optical density at 540 nm (BMG Omega, 77799 Ortenberg, Germany) was measured, and the results were expressed as a percentage compared to control cells.

## 4. Conclusions

Silver and iron nanoparticles were obtained either by traditional synthesis or using an aqueous extract of *S. fallax* moss. Both types of AgNPs and non-stabilized FeNPs were close to spherical and sized up to 100 nm, while extract-stabilized FeNPs were lamellar and up to 50 nm in diameter and up to 20 nm in thickness. FT-IR and hyperspectral microscopy studies confirmed the modification of extract-stabilized nanoparticles with biomolecules derived from the Sphagnum extract. Both types of silver nanoparticles prevented the formation of microbial biofilms by *E. coli*, while FeNPs were inefficient. The cytotoxicity of silver particles to mammalian cells varied depending on the cell line used. FeNPs were non-toxic to hMSC cells and slightly toxic to A549 cells at a high concentration of 15 µg/mL. The non-toxic FeNPs could find applications in biomedical studies while AgNPs can be used as antimicrobial agents. Thus, due to similarity in properties, the extract-stabilized NPs obtained at room temperature in an environmentally-friendly way can replace their counterparts obtained by conventional methods in many useful applications.

## Data Availability

The data will be made available from the corresponding authors upon reasonable request.

## Supplementary Materials

The following supporting information can be downloaded at: https://www.mdpi.com/article/10.3390/ijms24119274/s1.
