# Supplementary material for: Comparative Characterization of Iron and Silver Nanoparticles: Extract-Stabilized and Classical Synthesis Methods
Source: Int J Mol Sci. 2023 May 25;24(11):9274. doi: 10.3390/ijms24119274 (PMC10252514; doi:10.3390/ijms24119274)
Supplement: Supplementary file 1 [file ijms-24-09274-s001.zip › ijms-2382640-supplementary.pdf]

International Journal of Molecular Sciences

Comparative Characterization of Iron and Silver Nanoparticles: Extract-stabilized and Classical Synthesis Methods

Farida Akhatova, Svetlana Konnova, Marina Kryuchkova, Svetlana Batasheva, Kristina Mazurova, Anna Vikulina, Dmitry Volodkin, Elvira Rozhina

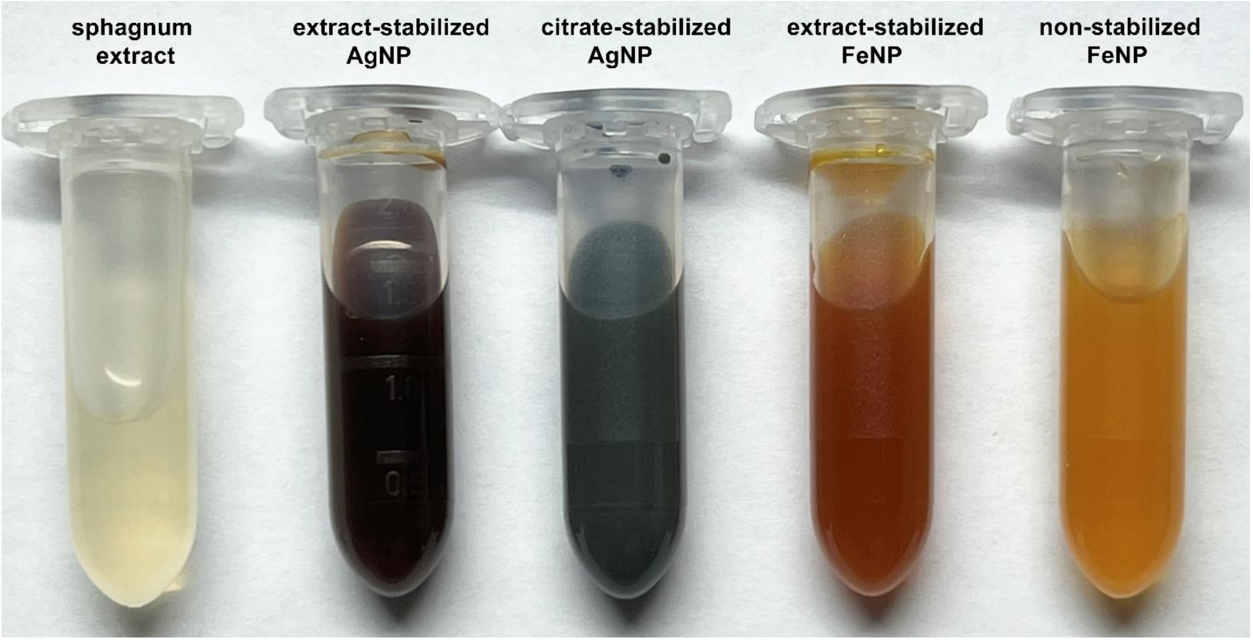

Figure S1. The images of the sphagnum extract and synthesized silver and iron nanoparticles.

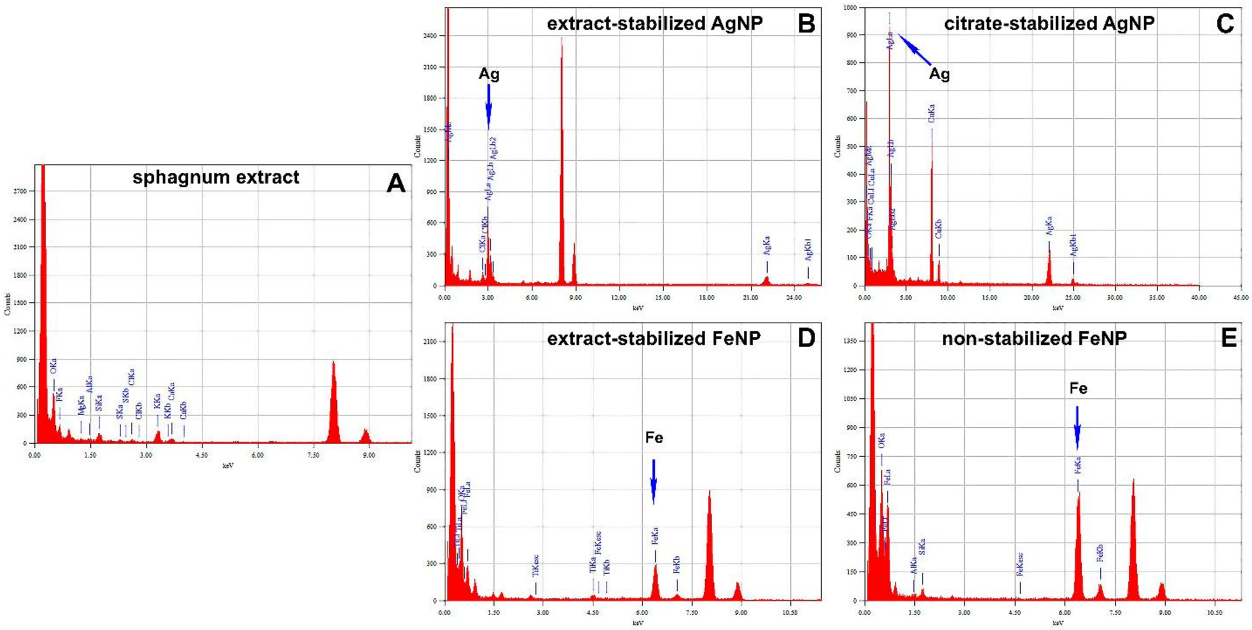

Figure S2. EDS spectrum of sphagnum extract (A), extract-stabilized AgNPs (B), citrate-stabilized AgNPs (C), extract-stabilized FeNPs (D), and non-stabilized FeNPs (E).

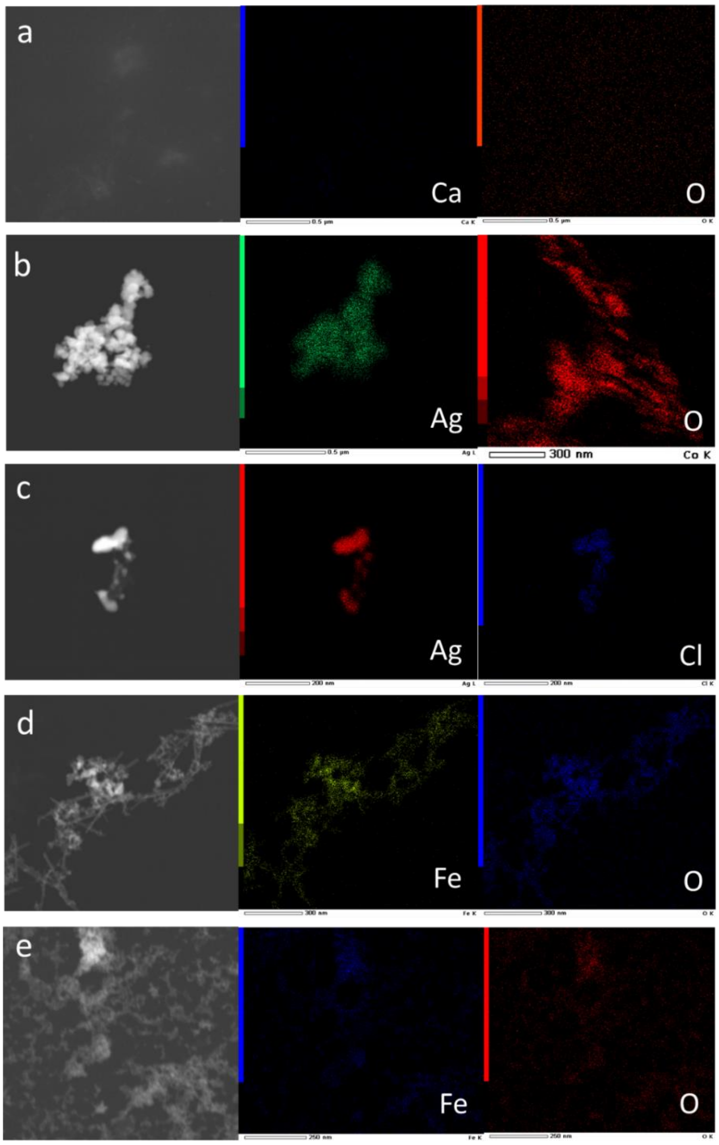

Figure S3. STEM images and elemental mapping of the Sphagnum extract (a), citrate-stabilized AgNPs (b), extract-stabilized AgNPs (c), non-stabilized FeNPs (d), extract-stabilized FeNPs (c).

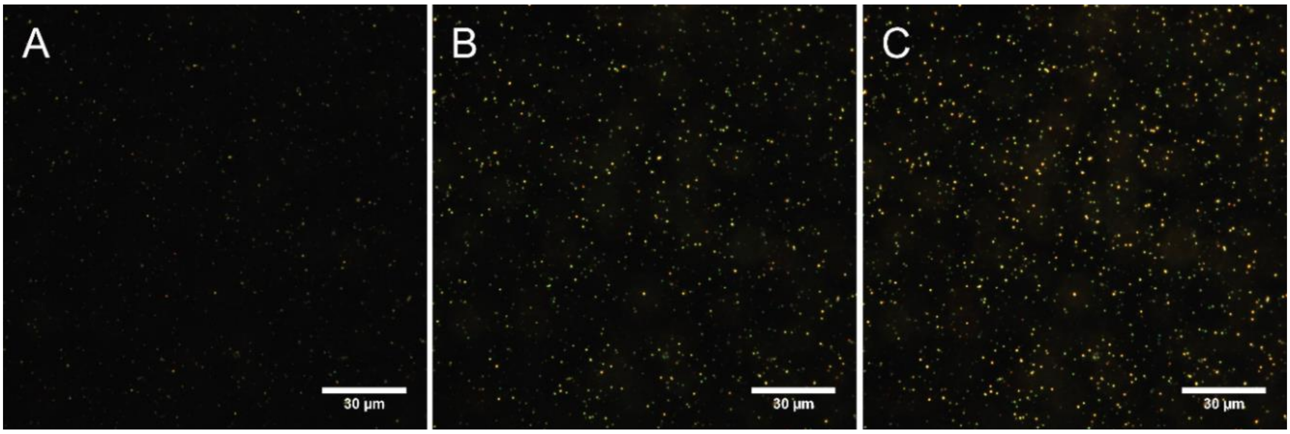

Figure S4. Dark-field images of extract-stabilized silver nanoparticles at 0 min (A), 10 min (B) and 20 min (C) from the beginning of observation.

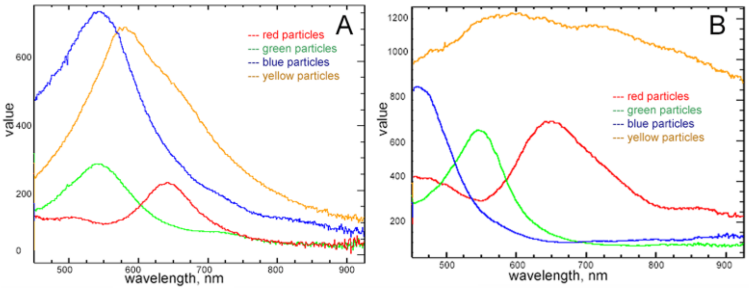

Figure S5. Hyperspectral profiles of extract-stabilized(A) and non-stabilized (B) silver nanoparticles of different colors.

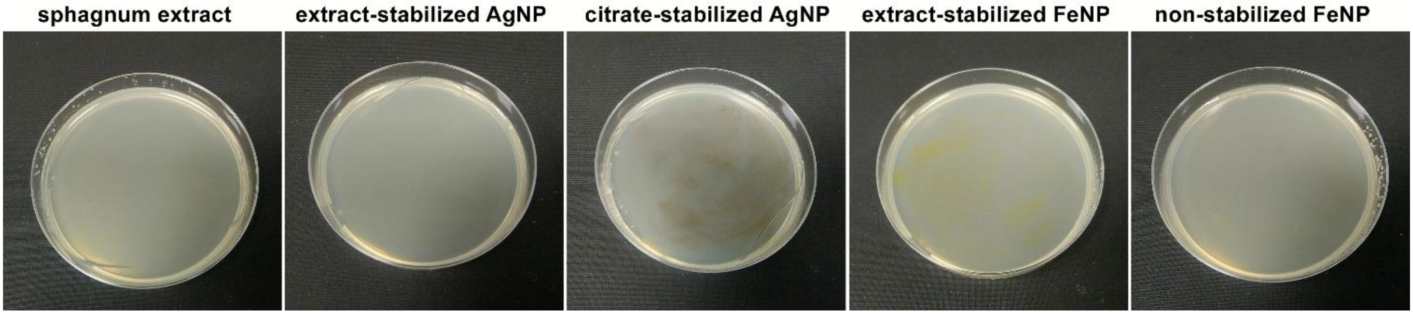

Figure S6. Petri dishes with nutrient medium inoculated with the Sphagnum extract or nanoparticles after 24h of cultivation.

Video S1. Video of the extract-stabilized nanoparticles maturation, recorded by a dark field microscope for 16 min.
